# The Maximal *C*^3^ Self-Complementary Trinucleotide Circular Code *X* in Genes of Bacteria, Archaea, Eukaryotes, Plasmids and Viruses

**DOI:** 10.3390/life7020020

**Published:** 2017-04-18

**Authors:** Christian J. Michel

**Affiliations:** Theoretical Bioinformatics, ICube, University of Strasbourg, CNRS, 300 Boulevard Sébastien Brant, 67400 Illkirch, France; c.michel@unistra.fr; Tel.: +33-368854462

**Keywords:** circular code in genes, DNA genes, RNA genes, double-stranded genes, single-stranded genes

## Abstract

In 1996, a set X of 20 trinucleotides was identified in genes of both prokaryotes and eukaryotes which has on average the highest occurrence in reading frame compared to its two shifted frames. Furthermore, this set X has an interesting mathematical property as X is a maximal $C^{3}$ self-complementary trinucleotide circular code. In 2015, by quantifying the inspection approach used in 1996, the circular code X was confirmed in the genes of bacteria and eukaryotes and was also identified in the genes of plasmids and viruses. The method was based on the preferential occurrence of trinucleotides among the three frames at the gene population level. We extend here this definition at the gene level. This new statistical approach considers all the genes, i.e., of large and small lengths, with the same weight for searching the circular code X. As a consequence, the concept of circular code, in particular the reading frame retrieval, is directly associated to each gene. At the gene level, the circular code X is strengthened in the genes of bacteria, eukaryotes, plasmids, and viruses, and is now also identified in the genes of archaea. The genes of mitochondria and chloroplasts contain a subset of the circular code X. Finally, by studying viral genes, the circular code X was found in DNA genomes, RNA genomes, double-stranded genomes, and single-stranded genomes.

## 1. Introduction

Circular code is a mathematical structure of genes and genomes. This concept initially found for genes is extended for genomes (non-coding regions of eukaryotes) according to recent results. A circular code X is a set of words such that any motif from X, called X motif, allows it to retrieve, maintain, and synchronize the original (construction) frame.

The circular code X identified in the genes of bacteria, eukaryotes, plasmids, and viruses [1,2] contains the 20 following trinucleotides

(1) X= {AAC,AAT,ACC,ATC,ATT,CAG,CTC,CTG,GAA,GAC, GAG,GAT,GCC,GGC,GGT,GTA,GTC,GTT,TAC,TTC}

which allows it to both retrieve the reading frame with a window of 13 nucleotides (Figure 3 in [3]) and to code the 12 following amino acids

(2) {Ala, Asn, Asp, Gln, Glu, Gly, Ile, Leu, Phe, Thr, Tyr, Val}.

The current genetic code is not circular. Thus, it cannot retrieve the reading frame. The loss during evolution of this circular code property on the 4-letter alphabet {A,C,G,T} required a complex translation mechanism using 20 amino acids and proteins in current genomes.

X motifs from Equation (1) are identified in (i) genes “universally” [1,4]; (ii) tRNAs of prokaryotes and eukaryotes [3,5]; (iii) rRNAs of prokaryotes (16S) and eukaryotes (18S), in particular in the ribosome decoding center where the universally conserved nucleotides G530, A1492, and A1493 are included in the X motifs [3,6,7]; and (iv) genomes (non-coding regions of eukaryotes) [4,8].

The X motifs of maximal cardinality 20 (composition) in genes with the properties of the circular code, $C^{3}$ and complementary allow the two reading frames and the four shifted frames to be retrieved by pairing between DNAs-DNAs, DNAs-mRNAs, mRNAs-rRNAs, mRNAs-tRNAs, and rRNAs-tRNAs, as shown with a 3D visualization of the X motifs in the ribosome [3,6,7].

**Figure 1:**
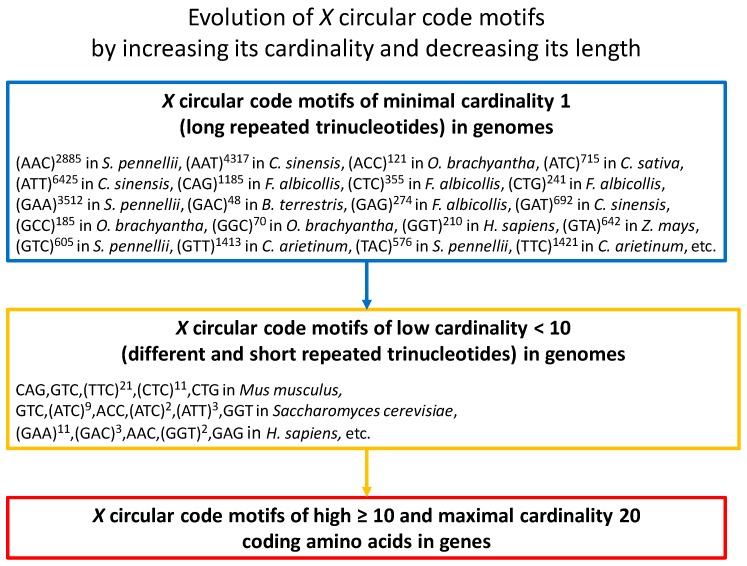
Model of evolution of the X circular code motifs (Equation (1)) by increasing its cardinality (composition) and decreasing its length. Evolution begins with X motifs of minimal cardinality 1 (long repeated trinucleotides) in genomes (the examples given are extracted from Table 2 in [8]). Then, the mutations in repeated trinucleotides lead to X motifs of low cardinality <10 (different and short repeated trinucleotides) in genomes (the examples given are extracted from Table 4 in [8]) up to X motifs of high ≥10 and maximal cardinality 20 coding the 12 amino acids (Equation (2)).

The X motifs in genomes have a different structure compared to the X motifs in genes [8]. Indeed, their cardinality is not maximal (less than 10 for an order of magnitude), their size is longer, and their structure contains repeated trinucleotides. Furthermore, the X motifs of minimal cardinality 1 generated with the 20 repeated trinucleotides $t^{n}$ where t∈X (Equation (1)) are very common in the genomes of eukaryotes (e.g., [8,9,10]). Their length n can be very large (e.g., n>6000, see Figure 1). The repeated trinucleotides are very unstable with mutation rates up to 100,000 times higher than the genomic average mutation rate. Mutation in repeats increases its evolutionary stability.

A model of evolution of the X motifs in genes and genomes can be proposed according to the previous works and the recent results [8]. It proposes that the X motifs of maximal cardinality 20 in genes have evolved from the X motifs of minimal cardinality 1 (repeated trinucleotides) in genomes (Figure 1). An X motif of minimal cardinality 1 which is unstable, mutates into an X motif of low cardinality <10 containing thus different repeated trinucleotides of short lengths. This evolutionary process continues by increasing the cardinality and decreasing the length of the X motifs up to generate the X motifs of high ≥10 and maximal cardinality 20 coding the 12 amino acids (Equation (2)) in genes. The X motifs of high cardinality have acquired the protein coding function in addition to the reading frame retrieval. This model suggests that the property of reading frame retrieval has preceded the protein coding function.

Since 1996, all the statistical analyses studying the preferential occurrence of trinucleotides among the three frames were done at the gene population level (kingdoms, taxonomic groups, genomes). We extend here the method from [1] at the gene level. This new approach is important as all the genes, i.e., of large and small lengths, are now considered with the same weight in the statistical definition for searching the circular code X. As a consequence, the concept of circular code, in particular the reading frame retrieval, is directly associated to each gene. Thus, at the gene level, the circular code X is searched here in the genes of bacteria, archaea, eukaryotes, plasmids, viruses, and eukaryotic organelles, i.e., mitochondria and chloroplasts. Finally, genes of double-stranded DNA and RNA viruses, and single-stranded DNA and RNA viruses are also analysed with this approach in order to assign a genetic information unit (DNA or RNA, double-stranded or single-stranded) to the circular code X.

## 2. Method

### 2.1. Definitions

We recall a few definitions without detailed explanations (i.e., without figures and examples) for understanding the main properties of the trinucleotide circular code X identified in genes [1,2].

**Notation** **1.** *Let us denote the nucleotide 4-letter alphabet* B={A,C,G,T} *where A stands for Adenine, C stands for Cytosine, G stands for Guanine, and T stands for Thymine. The trinucleotide set over* B *is denoted by* $B^{3}=\left\{AAA,\ldots ,TTT\right\}$ *. The set of non-empty words (words, respectively) over* B *is denoted by* $B^{+}$ *(*$B^{*}$, respectively).

**Notation** **2.** *Genes have three frames* f*. By convention here, the reading frame* f=0 *is set up by a start trinucleotide* {ATG,CTG,GTG,TTG}*, and the frames* f=1 *and* f=2 *are the reading frame* f=0 *shifted by one and two nucleotides in the* $5^{\prime }-3^{\prime }$ *direction (to the right), respectively.*

Two biological maps are involved in gene coding.

**Definition** **1.** *According to the complementary property of the DNA double helix, the nucleotide complementarity map* C:B→B *is defined by* C(A)=T*,* C(C)=G*,* C(G)=C*, and* C(T)=A*. According to the complementary and antiparallel properties of the DNA double helix, the trinucleotide complementarity map* $C:B^{3}\to B^{3}$ *is defined by* $C\left(l_{0}l_{1}l_{2}\right)=C\left(l_{2}\right)C\left(l_{1}\right)C\left(l_{0}\right)$ *for all* $l_{0},l_{1},l_{2}\in B$*. By extension to a trinucleotide set* S*, the set complementarity map* $C:\mathbb{P}\left(B^{3}\right)\to \mathbb{P}\left(B^{3}\right)$*,* ℙ *being the set of all subsets of* $B^{3}$*, is defined by* $C\left(S\right)=\left\{v\;:u,v\in B^{3},u\in S,v=C\left(u\right)\right\}$*, e.g.,* C({CGA, GAT})={ATC,TCG}*.*

**Definition** **2.** *The trinucleotide circular permutation map* $P:B^{3}\to B^{3}$ *is defined by* $P\left(l_{0}l_{1}l_{2}\right)=l_{1}l_{2}l_{0}$ *for all* $l_{0},l_{1},l_{2}\in B$*.* $P^{2}$ *denotes the 2nd iterate of* P*. By extension to a trinucleotide set* S*, the set circular permutation map* $P:\mathbb{P}\left(B^{3}\right)\to \mathbb{P}\left(B^{3}\right)$ *is defined by* $P\left(S\right)=\left\{v\;:u,v\in B^{3},u\in S,v=P\left(u\right)\right\}$*, e.g.,* P({CGA, GAT})={ATG,GAC} *and* P({CGA, GAT})={ACG,TGA} *.*

**Definition** **3.** *A set* $S\subseteq B^{+}$ *is a code if, for each* $x_{1},\ldots ,x_{n},y_{1},\ldots ,y_{m}\in S$*,* n,m≥1*, the condition* $x_{1}\ldots x_{n}=y_{1}\ldots y_{m}$ *implies* n=m *and* $x_{i}=y_{i}$ *for* i=1,…,n*.*

**Definition** **4.** *Any non-empty subset of the code* $B^{3}$ *is a code and called trinucleotide code* C*.*

**Definition** **5.** *A trinucleotide code* $C\subseteq B^{3}$ *is self-complementary if, for each* t∈C*,* C(t)∈C*, i.e.,* C=C(C)*.*

**Definition** **6.** *A trinucleotide code* $X\subseteq B^{3}$ *is circular if, for each* $x_{1},\ldots ,x_{n},y_{1},\ldots ,y_{m}\in X$*,* n,m≥1*,* $r\in B^{*}$, $s\in B^{+}$*, the conditions* $sx_{2}\ldots x_{n}r=y_{1}\ldots y_{m}$ *and* $x_{1}=rs$ *imply* n=m*,* r=ε *(empty word), and* $x_{i}=y_{i}$ *for* i=1,…,n*.*

The proofs to decide whether a code is circular or not are based on the flower automaton [2], the necklace 5*LDCN* (Letter Diletter Continued Necklace) [11], the necklace nLDCCN (Letter Diletter Continued Closed Necklace) with n∈{2,3,4,5} [12], and the graph theory [13].

**Definition** **7.** *A trinucleotide circular code* $X\subseteq B^{3}$ *is* $C^{3}$ *self-complementary if* X*,* $X_{1}=P\left(X\right)$*, and* $X_{2}=P^{2}\left(X\right)$ *are trinucleotide circular codes such that* X=C(X) *(self-complementary),* $C\left(X_{1}\right)=X_{2}$*, and* $C\left(X_{2}\right)=X_{1}$ *(*$X_{1}$ *and* $X_{2}$ *are complementary).*

The trinucleotide set $X=X_{0}$ (Equation (1)) coding the reading frame (f=0) in genes is a maximal (20 trinucleotides) $C^{3}$ self-complementary trinucleotide circular code [2] where the circular code $X_{1}=P\left(X\right)$ coding the frame f=1 contains the 20 following trinucleotides

(3) $$X_{1}=\;\{\mathrm{AAG},\mathrm{ACA},\mathrm{ACG},\mathrm{ACT},\mathrm{AGC},\mathrm{AGG},\mathrm{ATA},\mathrm{ATG},\mathrm{CCA},\mathrm{CCG},\;\mathrm{GCG},\mathrm{GTG},\mathrm{TAG},\mathrm{TCA},\mathrm{TCC},\mathrm{TCG},\mathrm{TCT},\mathrm{TGC},\mathrm{TTA},\mathrm{TTG}\}$$

and the circular code $X_{2}=P^{2}\left(X\right)$ coding the frame f=2 contains the 20 following trinucleotides

(4) $$X_{2}=\;\{\mathrm{AGA},\mathrm{AGT},\mathrm{CAA},\mathrm{CAC},\mathrm{CAT},\mathrm{CCT},\mathrm{CGA},\mathrm{CGC},\mathrm{CGG},\mathrm{CGT},\;\mathrm{CTA},\mathrm{CTT},\mathrm{GCA},\mathrm{GCT},\mathrm{GGA},\mathrm{TAA},\mathrm{TAT},\mathrm{TGA},\mathrm{TGG},\mathrm{TGT}\}.$$

The trinucleotide circular codes $X_{1}$ and $X_{2}$ are related by the permutation map, i.e., $X_{2}=P\left(X_{1}\right)$ and $X_{1}=P^{2}\left(X_{2}\right)$, and by the complementary map, i.e., $X_{1}=C\left(X_{2}\right)$ and $X_{2}=C\left(X_{1}\right)$ [14].

Several classes of methods were developed for identifying the circular code X in genes over the last 20 years: frequency methods [2,15,16], correlation function [17], covering capability function [18], and occurrence probability of a complementary/permutation (CP) trinucleotide set at the gene population level [1].

The class of the 216 $C^{3}$ self-complementary trinucleotide circular codes (Definition 7; [2]; list given in Tables 4a, 5a, and 6a in [19]; [20]) is included in a larger class of codes C by relaxing the circularity property which was defined in [1]:

**Definition** **8.** *A trinucleotide code* $C\subseteq B^{3}$ *is* $C^{3}$ *self-complementary if* C=C(C) *(self-complementary),* $C\left(C_{1}\right)=C_{2}$*, and* $C\left(C_{2}\right)=C_{1}$ *(*$C_{1}$ *and* $C_{2}$ *are complementary) where* $C_{1}=P\left(C\right)$ *and* $C_{2}=P^{2}\left(C\right)$ *.*

The statistical approach developed analyses the $C^{3}$ self-complementary codes (Definition 8) for searching the particular circular code X.

### 2.2. Gene Kingdoms

Gene kingdoms K of bacteria B, archaea A, plasmids ℙ, eukaryotes E, chromosomes of eukaryotes $E_{\mathrm{chr}}$, mitochondria M, chloroplasts ℂ, viruses V, and its five taxonomic double-stranded DNA viruses $V_{\mathrm{dsDNA}}$, double-stranded RNA viruses $V_{\mathrm{dsRNA}}$, single-stranded DNA viruses $V_{\mathrm{ssDNA}}$, single-stranded RNA viruses $V_{\mathrm{ssRNA}}$, and retro-transcribing viruses $V_{\mathrm{rt}}$ are obtained from the GenBank database (http://www.ncbi.nlm.nih.gov/genome/browse/, May 2016) (Table 1). Computer tests exclude genes when (i) their nucleotides do not belong to the alphabet B; (ii) they do not begin with a start trinucleotide {ATG,CTG,GTG,TTG}; (iii) they do not end with a stop trinucleotide {TAA,TAG,TGA}; and (iv) their lengths are not modulo 3. In order to have an order of magnitude of data acquisition (details in Table 1), the kingdom of bacteria B contains 15,735,053 genes and 5,222,267,667 trinucleotides (7,851,762 genes and 2,481,566,882 trinucleotides in [1]), i.e., a trinucleotide increase of about 110%, and the kingdom of eukaryotes E contains 4,356,391 genes and 2,406,844,838 trinucleotides (1,662,579 genes and 824,825,761 trinucleotides in [1]), i.e., a trinucleotide increase of about 192%. The gene kingdoms M, ℂ, $V_{\mathrm{dsRNA}}$, $V_{\mathrm{ssDNA}}$, and $V_{\mathrm{rt}}$ have gene and trinucleotide data that are significantly lower (less than 1 million trinucleotides) than the other gene kingdoms (Table 1).

### 2.3. Preferential Frame of a Trinucleotide in a Gene

The method developed in [1] for identifying the circular code X in genes determined the preferential frame of trinucleotides at the gene population level (kingdoms, taxonomic groups, genomes), i.e., after summing the trinucleotide frequencies of all genes in a kingdom. We extend this method at the gene level, i.e., the preferential frame of trinucleotides among the three frames is determined for each gene. There is no sum of trinucleotide frequencies of all genes in a kingdom. Thus, all the genes, i.e., of large and small lengths, have the same weight in respect to the preferential frame.

Consider a gene kingdom K listed in Table 1. Let $Pr_{f}\left(t,g\right)$ be the occurrence frequency of a trinucleotide $t\in B^{3}$ in a frame f∈{0,1,2} of a gene g belonging to a kingdom K. Thus, there are 3×64=192 trinucleotide occurrence frequencies $Pr_{f}\left(t,g\right)$ in the three frames f of a gene g. Then, the preferential frame F(t,g)∈{0,1,2} of a trinucleotide t in a gene g is the frame of maximal occurrence frequency $Pr_{f}\left(t,g\right)$ among the three frames f of g

(5) $$F\left(t,g\right)=\arg\underset{f\in \left\{0,1,2\right\}}{\max}Pr_{f}\left(t,g\right).$$

The three frequencies of a given trinucleotide are computed in the three frames 0, 1, and 2 of a gene. Then, the preferential frame of the trinucleotide in this gene is the frame associated to its highest trinucleotide frequency.

**Remark** **1.** *In* [1]*, the three occurrence frequencies* $Pr_{f}\left(t,K\right)$ *of a trinucleotide* t *in the three frames* f *computed in a gene kingdom* K*, always have different values, thus a unique preferential frame can be assigned to the trinucleotide. At the gene level, particularly for genes* g *of small lengths, a trinucleotide* t *may have an identical occurrence frequency* $Pr_{f}\left(t,g\right)$ *in two or three frames* f*. In this case, two or three preferential frames* F(t,g) *are assigned to the trinucleotide* t*. If a trinucleotide* t *is absent in a gene* g*, mainly for genes* g *of very small lengths, then no preferential frame is attributed to* t*.*

The indicator function $\delta_{f}\left(F\left(t,g\right)\right)\in \left\{0,1\right\}$ is 1 if the preferential frame F(t,g) of a trinucleotide t is equal to the frame f of a gene g, and 0 otherwise

(6) $$\delta_{f}\left(F\left(t,g\right)\right)=\{\begin{array}{c} 1\;\mathrm{if}\;F\left(t,g\right)=f\\ 0\;\mathrm{otherwise} \end{array}$$

where F(t,g) is defined in Equation (5).

### 2.4. Number of Preferential Frames of a Trinucleotide in a Gene Kingdom

The number $Nb_{f}\left(t,K\right)\in \mathbb{N}$ of preferential frames of a trinucleotide $t\in B^{3}$ for each frame f∈{0,1,2} in a gene kingdom K is simply obtained by summing for all genes in K

(7) $$Nb_{f}\left(t,K\right)=\underset{g\in K}{\sum }\delta_{f}\left(F\left(t,g\right)\right)$$

where $\delta_{f}\left(F\left(t,g\right)\right)$ is defined in Equation (6).

### 2.5. Occurrence Probability of a Complementary/Permutation Trinucleotide Set in a Gene Kingdom

In order to study the $C^{3}$ self-complementary codes C (Definition 8) including the class of circular codes, and in particular the circular code X, Equation (7) for a trinucleotide t is expanded to a set T of six trinucleotides involving the complementarity map C and the permutation map P simultaneously, precisely $T=\left\{T^{0},T^{1},T^{2}\right\}$ with $T^{0}=\left\{t,C\left(t\right)\right\}$ in frame 0, $T^{1}=P(T^{0})=\left\{P\left(t\right),P(C(t))\right\}$ in frame 1, $T^{2}=P^{2}(T^{0})=\left\{P^{2}\left(t\right),P^{2}(C(t))\right\}$ in frame 2, and $t\in B^{3}\backslash \left\{\mathrm{AAA},\mathrm{CCC},\mathrm{GGG},\mathrm{TTT}\right\}$. T is called a complementary and permutation (CP) trinucleotide set and is completely defined by the trinucleotide t.

**Remark** **2.** $P\left(t\right)=C(P^{2}(C(t)))$ and $P^{2}\left(t\right)=C(P(C(t)))$ *(proof obvious)*.

When the trinucleotide t is given then the trinucleotide C(t) is also known. Thus, there are 60/2=30 CP trinucleotide sets noted $T_{1},\ldots ,T_{30}$ where $T_{i}=\left\{T_{i}^{0},T_{i}^{1},T_{i}^{2}\right\}$ with $T_{i}^{0}={\left\{t,C\left(t\right)\right\}}_{i}$ in frame 0, $T_{i}^{1}=P{\left(T^{0}\right)}_{i}={\left\{P\left(t\right),PC\left(t\right)\right\}}_{i}$ in frame 1, and $T_{i}^{2}=P^{2}{\left(T^{0}\right)}_{i}={\left\{P^{2}\left(t\right),P^{2}C\left(t\right)\right\}}_{i}$ in frame 2. A maximal (20 trinucleotides) $C^{3}$ self-complementary code C is identified with the first 10 values of the numbers $Nb\left(T_{1},K\right),\ldots ,Nb\left(T_{10},K\right)$ (defined below). Precisely, the code C has 20 trinucleotides $C=C_{0}=\left\{T_{1}^{0},\ldots ,T_{10}^{0}\right\}$ in frame 0, 20 trinucleotides $C_{1}=P\left(C\right)=\left\{T_{1}^{1},\ldots ,T_{10}^{1}\right\}$ in frame 1, and 20 trinucleotides $C_{2}=P^{2}\left(C\right)=\left\{T_{1}^{2},\ldots ,T_{10}^{2}\right\}$ in frame 2 with C=C(C) (self-complementary), $C\left(C_{1}\right)=C_{2}$, and $C\left(C_{2}\right)=C_{1}$ ($C_{1}$ and $C_{2}$ are complementary). There are $\left(\begin{array}{c} 30\\ 10 \end{array}\right)=30,045,015$ $C^{3}$ self-complementary trinucleotide codes, and among them only 216 are circular [2,20].

**Notation** **3.** *A* *CP trinucleotide set* $T=\left\{T^{0},T^{1},T^{2}\right\}$ *belongs to the* $C^{3}$ *self-complementary trinucleotide circular code* X*, i.e.,* T∈X*, if* $T^{0}\cap^{\text{​}}X\neq \emptyset$*, i.e., if the trinucleotide* t *and its complementary* *trinucleotide* C(t) *belong to* X*. Ten* *CP trinucleotide sets* T *among 30 belong to the* $C^{3}$ *circular code* X*, i.e., such that 10 sets* $T^{0}\in X$ *with* $T^{1}=P(T^{0})\in P(X)=X_{1}$ *and* $T^{2}=P^{2}(T^{0})\in P^{2}(X)=X_{2}$*.*

**Notation** **4.** *In order to facilitate the reading of* Table 2*, the* *30* *CP trinucleotide sets* $T=\left\{T^{0},T^{1},T^{2}\right\}$ *are presented in the following way (i) the first 10* *sets* $T_{1},\ldots ,T_{10}$ *belong to the circular code* X *(with* $T^{0}=\left\{t,C\left(t\right)\right\}\in X$*,* $T^{1}\in X_{1}$ *and* $T^{2}\in X_{2}$*) and are in lexicographical order with respect to the trinucleotide* t∈X *(in bold),* *and (ii) the 20 remaining sets* $T_{11},\ldots ,T_{30}$ *are in lexicographical order with respect to the trinucleotide* $t\in X_{1}$ *(in italics).*

The occurrence number Nb(T,K) of a CP trinucleotide set $T=\left\{T^{0},T^{1},T^{2}\right\}$ in a gene kingdom K is equal to

(8) $$Nb\left(T,K\right)=Nb_{0}\left(t,K\right)+Nb_{0}\left(C\left(t\right),K\right)+Nb_{1}\left(P\left(t\right),K\right)+Nb_{1}\left(P\left(C\left(t\right)\right),K\right)+Nb_{2}\left(P^{2}\left(t\right),K\right)+Nb_{2}\left(P^{2}\left(C\left(t\right)\right),K\right)$$

where $Nb_{f}\left(t,K\right)$ is defined in Equation (7).

In order to normalize the numbers Nb(T,K) which depend on the numbers of genes in a kingdom K, we simply define the occurrence probability Pb(T,K) of a CP trinucleotide set $T=\left\{T^{0},T^{1},T^{2}\right\}$ in a gene kingdom K as follows

(9) $$Pb\left(T,K\right)=\frac{Nb\left(T,K\right)}{\sum_{i=1}^{30}Nb\left(T_{i},K\right)}$$

where Nb(T,K) is defined in Equation (8).

The parameter Rk(T,K)∈{1,…,30} gives the rank of the values Pb(T,K) among the 30 CP trinucleotide sets T, the 1st rank being associated to the highest value of Pb(T,K) and the 30th rank, to the lowest value of Pb(T,K).

### 2.6. A Statistical Test to Evaluate the Significance of the Obtained Ranks

In order to evaluate the statistical significance of the ranks Rk(T,K) of the probabilities Pb(T,K) (Equation (9)) of the 30 CP trinucleotide sets T in a given kingdom K, we derive confidence intervals for Pb(T,K). If the confidence interval for two probabilities Pb(T,K) do not overlap, then their associated ranks Rk(T,K) are assumed to be valid (in the population). The confidence interval for two probabilities Pb(T,K) is evaluated by using the classical 2-sample *z*-test which is briefly recalled here.

Let P(T) and $P(T^{\prime })$ be the populations associated to the CP trinucleotide sets T and $T^{\prime }$ of probabilities Pb(T,P) and $Pb\left(T^{\prime },P\right)$, respectively. The probabilities Pb(T,K) and $Pb\left(T^{\prime },K\right)$ of T and $T^{\prime }$ are observed in a given gene kingdom K (sample) of size $n=\sum_{i=1}^{30}Nb\left(T_{i},K\right)$ (defined from Equation (8)). The tests carried out in Section 3 are applied on large samples (the size of the smallest sample analysed being n=10921 with the archaea A). Thus, the assumptions of normality for the variables and of the homogeneity for the variances in the two populations are not needed. The equality $H_{0}:Pb\left(T,P\right)\geq Pb\left(T^{\prime },P\right)$ is tested against the alternative $H_{1}:Pb\left(T,P\right)<Pb\left(T^{\prime },P\right)$ if they are not equal. Under $H_{0}$ and with large samples (n>30), $\min\{nPb\left(T,P\right),n(1-Pb\left(T,P\right)),nPb\left(T^{\prime },P\right),n(1-Pb\left(T^{\prime },P\right))\}>5$ (always verified in the tests carried out in Section 3), and T and $T^{\prime }$ are independent events (realistic hypothesis with kingdoms K of large sizes), then

$$Z=\frac{Pb\left(T,P\right)-Pb\left(T^{\prime },P\right)}{\sqrt{\left(\frac{nPb\left(T,P\right)+nPb\left(T^{\prime },P\right)}{n+n}\right)\left(1-\frac{nPb\left(T,P\right)+nPb\left(T^{\prime },P\right)}{n+n}\right)\left(\frac{1}{n}+\frac{1}{n}\right)}}=\frac{\sqrt{2}\left(Pb\left(T,P\right)-Pb\left(T^{\prime },P\right)\right)}{\sqrt{-\frac{\left(Pb\left(T,P\right)+Pb\left(T^{\prime },P\right)-2\right)\left(Pb\left(T,P\right)+Pb\left(T^{\prime },P\right)\right)}{n}}}\sim N\left(0,1\right).$$

The *z*-value and the *p*-value are given for each statistical test carried out in Section 3.

### 2.7. Explained Example of the Statistical Approach Developed

As an example, we explain the definition of the occurrence probability Pb(T,K) (Equation (9)) which takes the value of 6.1% (see Table 2) with the CP trinucleotide set $T_{1}=\left\{T_{1}^{0},T_{1}^{1},T_{1}^{2}\right\}$ with $T_{1}^{0}=\left\{t,C\left(t\right)\right\}=\left\{\mathrm{AAC},\mathrm{GTT}\right\}$ in frame 0, $T_{1}^{1}=P{\left(T^{0}\right)}_{1}=\left\{P(t),P(C\left(t\right))\right\}=\left\{\mathrm{ACA},\mathrm{TTG}\right\}$ in frame 1 and $T_{1}^{2}=P^{2}{\left(T^{0}\right)}_{1}=\left\{P^{2}(t),P^{2}(C\left(t\right))\right\}=\left\{\mathrm{CAA},\mathrm{TGT}\right\}$ in frame 2 in the gene kingdom of bacteria K=B (Table 1).

The 3×64=192 occurrence frequencies $Pr_{f}\left(t,g\right)$ of the 64 trinucleotides t are computed in the three frames f of each gene g belonging to B. Then, the preferential frame F(t,g) of each trinucleotide t for each gene g in B is determined according to Equation (5). For example, with the trinucleotide t=AAC in a gene $g_{1}$ of B, if the frequency $Pr_{0}\left(\mathrm{AAC},g_{1}\right)$ of AAC in frame f=0 (reading frame) is greater than the two frequencies $Pr_{1}\left(\mathrm{AAC},g_{1}\right)$ and $Pr_{2}\left(\mathrm{AAC},g_{1}\right)$ of AAC in frames f=1 and f=2, i.e., $Pr_{0}\left(\mathrm{AAC},g_{1}\right)>Max\left\{Pr_{1}\left(\mathrm{AAC},g_{1}\right),Pr_{2}\left(\mathrm{AAC},g_{1}\right)\right\}$, then the preferential frame of AAC in $g_{1}$ is 0, i.e., $F\left(\mathrm{AAC},g_{1}\right)=0$.

The indicator function $\delta_{f}\left(F\left(t,g\right)\right)$ of each trinucleotide t for each gene g in B is obtained from Equation (6). With the previous example of AAC in the gene $g_{1}$ of B, the indicator function is equal to $\delta_{0}\left(F\left(\mathrm{AAC},g_{1}\right)\right)=1$ for the frame f=0 and $\delta_{1}\left(F\left(\mathrm{AAC},g_{1}\right)\right)=\delta_{2}\left(F\left(\mathrm{AAC},g_{1}\right)\right)=0$ for the frames f=1 and f=2.

The number $Nb_{f}\left(t,B\right)$ of preferential frames of each trinucleotide t for each frame f in B is computed according to Equation (7). With the previous example of AAC in B, the following numbers are obtained: $Nb_{0}\left(\mathrm{AAC},B\right)=3486$ for the frame f=0, $Nb_{1}\left(\mathrm{AAC},B\right)=1742$ for the frame f=1, and $Nb_{2}\left(\mathrm{AAC},B\right)=1819$ for the frame f=2. Thus, the preferential frame of AAC in B is 0.

The occurrence number Nb(T,B) of the 30 CP trinucleotide sets $T_{i}=\left\{T_{i}^{0},T_{i}^{1},T_{i}^{2}\right\}$ in B is determined according to Equation (8). With $T_{1}$ in B, the following numbers are obtained: $Nb_{0}\left(\mathrm{GTT},B\right)=3765$ for the frame f=0, $Nb_{1}\left(\mathrm{ACA},B\right)=4002$ and $Nb_{1}\left(\mathrm{TTG},B\right)=5650$ for the frame f=1, and $Nb_{2}\left(\mathrm{CAA},B\right)=3999$ and $Nb_{2}\left(\mathrm{TGT},B\right)=4677$ for the frame f=2. Then, the occurrence number of $T_{1}$ in B is equal to $Nb\left(T_{1},B\right)=Nb_{0}\left(\mathrm{AAC},B\right)+Nb_{0}\left(\mathrm{GTT},B\right)+Nb_{1}\left(\mathrm{ACA},B\right)+Nb_{1}\left(\mathrm{TTG},B\right)+Nb_{2}\left(\mathrm{CAA},B\right)+Nb_{2}\left(\mathrm{TGT},B\right)=3486+3765+4002+5650+3999+4677=25579$.

Finally, the occurrence probability Pb(T,B) of the 30 CP trinucleotide sets $T_{i}=\left\{T_{i}^{0},T_{i}^{1},T_{i}^{2}\right\}$ in B is deduced from Equation (9). With $T_{1}$ in B, the occurrence probability of $T_{1}$ in B is equal to $Pb\left(T_{1},B\right)=\frac{Nb\left(T_{1},B\right)}{\sum_{i=1}^{30}Nb\left(T_{i},B\right)}=\frac{Nb\left(T_{1},B\right)}{Nb\left(T_{1},B\right)+...+Nb\left(T_{30},B\right)}=\frac{25579}{25579+...+11856}=\frac{25579}{422598}\approx 6.1\%$.

## 3. Results

### 3.1. Maximal $C^{3}$ Self-Complementary Circular Code X in Genes

This new statistical approach will show that the same set X of 20 trinucleotides among $\left(\begin{array}{c} 30\\ 10 \end{array}\right)=30,045,015$ sets occurs preferentially in genes (reading frame) of bacteria B, archaea A, plasmids ℙ, eukaryotes E, and viruses V. This set X is the maximal $C^{3}$ self-complementary circular code defined in Equation (1).

#### 3.1.1. Circular Code X in Genes of Bacteria

In the genes of bacteria B, the 10 CP trinucleotide sets $T_{1},\ldots ,T_{10}\in X$ have occurrence probabilities Pb(T,B) (Equation (9)) with the 10 highest ranks Rk(T,B) among 30 (Table 2), i.e., {t,C(t)}∈X, $\left\{P(t),P(C\left(t\right))\right\}\in X_{1}$ and $\left\{P^{2}(t),P^{2}(C\left(t\right))\right\}\in X_{2}$ leading to the 20 trinucleotides of X in frame 0, 20 trinucleotides of $X_{1}$ in frame 1, and 20 trinucleotides of $X_{2}$ in frame 2. The highest rank with $Pb\left(T_{8},B\right)=8.2\%$ is related to the complementary pair {t,C(t)}={GAC,GTC}∈X. The 10th rank with $Pb\left(T_{5},B\right)=4.55\%$ is very significantly greater than the 11th rank with $Pb\left(T_{22},B\right)=3.31\%$ ($n=\sum_{i=1}^{30}Nb\left(T_{i},B\right)=422598$, z-value=29.33, $p\mathrm{-value}=10^{-189}$). The 20 trinucleotides of the circular code X are identified in the genes of bacteria:

(10) $$X_{B}=\;X.$$

The same result is obtained at the gene level and the gene population level [1].

#### 3.1.2. Circular Code X in Genes of Archaea

In the genes of archaea A, the eight CP trinucleotide sets $T_{1},T_{2},T_{4},T_{6},\ldots ,T_{10}\in X$ (except $T_{3}$ and $T_{5}$) have occurrence probabilities Pb(T,A) with the eight highest ranks Rk(T,A) among 30 (Table 2). The highest rank with $Pb\left(T_{8},A\right)=9.7\%$ is also related to the complementary pair {GAC,GTC}∈X. The CP set $T_{22}\notin X$ with $Rk\left(T_{22},A\right)=9$ explains that the two complementary trinucleotides {t,C(t)}={ACC,GGT}∈X ($T_{3}$) do not occur preferentially in A. As the CP set $T_{5}\in X$ has a rank $Rk\left(T_{5},A\right)=13$ with $Pb\left(T_{5},A\right)=3.66\%$ greater than $Rk\left(T_{15},A\right)=14$ with $Pb\left(T_{15},A\right)=3.39\%$ and $Rk\left(T_{28},A\right)=15$ with $Pb\left(T_{28},A\right)=2.95\%$, the two complementary trinucleotides {t,C(t)}={CAG,CTG}∈X occur preferentially in A compared to {AGC,GCT} ($T_{15}$) and {GCA,TGC} ($T_{28}$), however the statistical significance between the ranks $Rk\left(T_{5},A\right)$ and $Rk\left(T_{15},A\right)$ is not confirmed due to the lack of archaeal gene data (see Section 2.2) ($n=\sum_{i=1}^{30}Nb\left(T_{i},A\right)=10921$, z-value=1.08, p-value=0.14). Thus, a subset of X of 18 trinucleotides (a non-maximal $C^{3}$ self-complementary circular code) is identified in the genes of archaea:

(11) $$X_{A}=\;XY_{A}\;\mathrm{with}\;Y_{A}=\left\{\mathrm{ACC},\mathrm{GGT}\right\}.$$

Note that the code $X_{A}\cup^{\text{​}}\left\{\mathrm{CAC},\mathrm{GTG}\right\}$ ($T_{22}$) is the variant X code observed in *Deinococcus* [1]. The circular code X retrieved in the genes of archaea is a new result which was not found in a study of variant X codes in archaeal genomes [15].

#### 3.1.3. Circular Code X in Genes of Plasmids

In the genes of plasmids ℙ, the 10 CP trinucleotide sets $T_{1},\ldots ,T_{10}\in X$ have occurrence probabilities Pb(T,ℙ) with the 10 highest ranks Rk(T,ℙ) among 30 (Table 2). The highest rank with $Pb\left(T_{8},\mathbb{P}\right)=7.8\%$ is again related to the complementary pair {GAC,GTC}∈X. The 10th rank with $Pb\left(T_{5},\mathbb{P}\right)=3.93\%$ is very significantly greater than the 11th rank with $Pb\left(T_{21},\mathbb{P}\right)=3.43\%$ ($n=\sum_{i=1}^{30}Nb\left(T_{i},\mathbb{P}\right)=144366$, z-value=7.14, $p\mathrm{-value}=10^{-13}$). The 20 trinucleotides of the circular code X are identified in the genes of plasmids:

(12) $$X_{\mathbb{P}}=\;X.$$

The same result is obtained at the gene level and the gene population level [1].

#### 3.1.4. Circular Code X in Genes of Eukaryotes

In the genes of eukaryotes E, the 10 CP trinucleotide sets $T_{1},\ldots ,T_{10}\in X$ have occurrence probabilities Pb(T,E) with the 10 highest ranks Rk(T,E) among 30 (Table 2). The highest rank with $Pb\left(T_{8},E\right)=9.0\%$ is again related to the complementary pair {GAC,GTC}∈X. The 10th rank with $Pb\left(T_{5},E\right)=4.23\%$ is significantly greater than the 11th rank with $Pb\left(T_{22},E\right)=3.82\%$ ($n=\overset{30}{\underset{i=1}{\sum }}Nb\left(T_{i},E\right)=11401$, z-value=1.57, p-value=0.06). The 20 trinucleotides of the circular code X are identified in the genes of eukaryotes:

(13) $$X_{E}=\;X.$$

The same result is obtained at the gene level and the gene population level [1].

The subset $X_{E_{Homo\;sapiens}}=\;X\backslash \left\{\mathrm{ACC},\mathrm{GCC},\mathrm{GGC},\mathrm{GGT}\right\}$ of X of 16 trinucleotides in the genes of *Homo sapiens* identified at the gene level is also identical to the subset found at the gene population level [1].

#### 3.1.5. Circular Code X in Genes of Eukaryotic Chromosomes

The statistical analysis in Section 3.1.4 takes the eukaryotic genome as the genetic information unit. Indeed, Equation (7) with g∈E is achieved with Card(E)=190 eukaryotic genomes (see Table 1). We complete this classical approach by choosing the eukaryotic chromosome as the genetic information unit. Thus, Equation (7) with $g\in E_{\mathrm{chr}}$ is performed with $Card\left(E_{\mathrm{chr}}\right)=2979$ eukaryotic chromosomes of Card(E)=190 genomes (see Table 1).

In the genes of eukaryotic chromosomes $E_{\mathrm{chr}}$, the 10 CP trinucleotide sets $T_{1},\ldots ,T_{10}\in X$ have occurrence probabilities $Pb\left(T,E_{\mathrm{chr}}\right)$ with the 10 highest ranks $Rk\left(T,E_{\mathrm{chr}}\right)$ among 30 (Table 2). The highest rank with $Pb\left(T_{8},E_{\mathrm{chr}}\right)=9.1\%$ is again related to the complementary pair {GAC,GTC}∈X. The 10th rank with $Pb\left(T_{3},E_{\mathrm{chr}}\right)=4.74\%$ is very significantly greater than the 11th rank with $Pb\left(T_{22},E_{\mathrm{chr}}\right)=4.47\%$ ($n=\sum_{i=1}^{30}Nb\left(T_{i},E_{\mathrm{chr}}\right)=179136$, z-value=3.86, $p\mathrm{-value}=10^{-5}$). The 20 trinucleotides of the circular code X are identified in the genes of eukaryotic chromosomes:

(14) $$X_{E_{\mathrm{chr}}}=\;X.$$

It is a new result which completes the statistical analysis of genes in eukaryotic genomes (Section 3.1.4).

#### 3.1.6. Non-Maximal Circular Code X in Genes of Eukaryotic Organelles

The genes of eukaryotic organelles, i.e., mitochondria and chloroplasts, are investigated with this statistical approach. It should also be stressed that the available data have an order of magnitude very significantly lower than the other gene kingdoms studied (less than 1 million trinucleotides for each class of organelles, see Table 1). However, we can already observe some statistical trends with the trinucleotides in the preferential frame.

##### Non-Maximal Circular Code X in Genes of Mitochondria

Surprisingly, in the genes of mitochondria M, the four CP trinucleotide sets $T_{9},T_{7},T_{8},T_{3}\in X$ have occurrence probabilities Pb(T,M) with the four highest ranks Rk(T,M) among 30 (Table 2). The CP set $T_{28}\notin X$ with $Rk\left(T_{28},M\right)=5$ explains that the two complementary trinucleotides {CAG,CTG}∈X ($T_{5}$) do not occur preferentially in M. The CP set $T_{25}\notin X$ with $Rk\left(T_{25},M\right)=6$ determines that the two complementary trinucleotides {CTC,GAG}∈X ($T_{6}$) do not occur preferentially in M. The CP set $T_{24}\notin X$ with $Rk\left(T_{24},M\right)=7$ implies that the two complementary trinucleotides {ATC,GAT}∈X ($T_{4}$) do not occur preferentially in M. The CP set $T_{17}\notin X$ with $Rk\left(T_{17},M\right)=11$ explains that the two complementary trinucleotides {AAT,ATT}∈X ($T_{2}$) do not occur preferentially in M. Thus, a subset of X of 12 trinucleotides (a non-maximal $C^{3}$ self-complementary circular code) is identified in the genes of mitochondria M:

(15) $$X_{M}=\;X\backslash Y_{M}\;\mathrm{with}\;Y_{M}=\left\{\mathrm{AAT},\mathrm{ATC},\mathrm{ATT},\mathrm{CAG},\mathrm{CTC},\mathrm{CTG},\mathrm{GAG},\mathrm{GAT}\right\}.$$

This subset $X_{M}=\;\left\{\mathrm{AAC},\mathrm{ACC},\mathrm{GAA},\mathrm{GAC},\mathrm{GCC},\mathrm{GGC},\mathrm{GGT},\mathrm{GTA},\mathrm{GTC},\mathrm{GTT},\mathrm{TAC},\mathrm{TTC}\right\}$ is very close to the subset $X_{\tilde{M}}=\;\left\{\mathrm{ACC},\mathrm{ATC},\mathrm{CTC},\mathrm{GAA},\mathrm{GAC},\mathrm{GAT},\mathrm{GCC},\mathrm{GGC},\;\mathrm{GGT},\mathrm{GTA},\mathrm{GTC},\mathrm{GTT},\mathrm{TTC}\right\}$ of X of 13 trinucleotides previously identified by inspection in mitochondrial genes [21], as $X_{M}⋂X_{\tilde{M}}=\left\{\mathrm{ACC},\mathrm{GAA},\mathrm{GAC},\mathrm{GCC},\mathrm{GGC},\mathrm{GGT},\mathrm{GTA},\mathrm{GTC},\mathrm{GTT},\mathrm{TTC}\right\}$ has 10 trinucleotides in common.

##### Non-Maximal Circular Code X in Genes of Chloroplasts

In the genes of chloroplasts ℂ, the highest occurrences of CP trinucleotide sets again belong to the circular code X. The three CP trinucleotide sets $T_{2},T_{9},T_{3}\in X$ have occurrence probabilities Pb(T,ℂ) with the three highest ranks Rk(T,ℂ) among 30 (Table 2). The CP set $T_{13}\notin X$ with $Rk\left(T_{13},\mathbb{C}\right)=4$ explains that the two complementary trinucleotides {GAC,GTC}∈X ($T_{8}$) do not occur preferentially in ℂ. The CP set $T_{28}\notin X$ with $Rk\left(T_{28},\mathbb{C}\right)=5$ states that the two complementary trinucleotides {CAG,CTG}∈X ($T_{5}$) do not occur preferentially in ℂ. The CP set $T_{14}\notin X$ with $Rk\left(T_{14},\mathbb{C}\right)=8$ implies that the two complementary trinucleotides {GTA,TAC}∈X ($T_{10}$) do not occur preferentially in ℂ. The CP set $T_{18}\notin X$ with $Rk\left(T_{18},\mathbb{C}\right)=10$ explains that the two complementary trinucleotides {ATC,GAT}∈X ($T_{4}$) do not occur preferentially in ℂ. The CP set $T_{25}\notin X$ with $Rk\left(T_{25},\mathbb{C}\right)=12$ implies that the two complementary trinucleotides {CTC,GAG}∈X ($T_{6}$) do not occur preferentially in ℂ. Thus, a subset of X of 10 trinucleotides (a non-maximal $C^{3}$ self-complementary circular code) is identified in the genes of chloroplasts ℂ:

(16) $$X_{\mathbb{C}}=\;X\backslash Y_{\mathbb{C}}\;\mathrm{with}\;Y_{\mathbb{C}}=\left\{\mathrm{ATC},\mathrm{CAG},\mathrm{CTC},\mathrm{CTG},\mathrm{GAC},\mathrm{GAG},\mathrm{GAT},\mathrm{GTA},\mathrm{GTC},\mathrm{TAC}\right\}.$$

#### 3.1.7. Circular Code X in Genes of Viruses

In the genes of viruses V, the nine CP trinucleotide sets $T_{1},\ldots ,T_{4},T_{6},\ldots ,T_{10}\in X$ (except $T_{5}$) have occurrence probabilities Pb(T,V) with the nine highest ranks Rk(T,V) among 30 (Table 2). The highest rank with $Pb\left(T_{8},V\right)=7.2\%$ is again related to the complementary pair {GAC,GTC}∈X. The CP set $T_{15}\notin X$ with $Rk\left(T_{15},V\right)=10$ explains that the two complementary trinucleotides {CAG,CTG}∈X ($T_{5}$) do not occur preferentially in V. Thus, a subset of X of 18 trinucleotides (a non-maximal $C^{3}$ self-complementary circular code) is identified in the genes of viruses:

(17) $$X_{V}=\;X\backslash Y_{V}\;\mathrm{with}\;Y_{V}=\left\{\mathrm{CAG},\mathrm{CTG}\right\}.$$

The statistical method of viral genes at the gene population level [1] could not decide between the two codes $X_{18}=X\backslash \left\{\mathrm{CAG},\mathrm{CTG}\right\}$ and $X_{16}=X\backslash \left\{\mathrm{CAG},\mathrm{CTG},\mathrm{GTA},\mathrm{TAC}\right\}$. The statistical analysis at the gene level confirms the code $X_{V}=\;X_{18}$ of 18 trinucleotides in the genes of viruses.

### 3.2. Circular Code X Found in DNA and RNA Genomes and in Double-Stranded and Single-Stranded Genomes

The self-complementary property of the circular code X has been related since 1996 to the complementary property of the DNA double helix. In order to deepen this idea, we searched with this statistical approach the circular code X in five important sub-classes of viral genes using either DNA genome or RNA genome, and either double-stranded genome or single-stranded genome, i.e., in the genes of double-stranded DNA viruses $V_{\mathrm{dsDNA}}$, double-stranded RNA viruses $V_{\mathrm{dsRNA}}$, single-stranded DNA viruses $V_{\mathrm{ssDNA}}$, single-stranded RNA viruses $V_{\mathrm{ssRNA}}$, and retro-transcribing viruses $V_{\mathrm{rt}}$.

In the genes of double-stranded DNA viruses $V_{\mathrm{dsDNA}}$, the 10 CP trinucleotide sets $T_{1},\ldots ,T_{10}\in X$ have occurrence probabilities $Pb\left(T,V_{\mathrm{dsDNA}}\right)$ with the 10 highest ranks $Rk\left(T,V_{\mathrm{dsDNA}}\right)$ among 30 (Table 2). Thus, the circular code X is found in $V_{\mathrm{dsDNA}}$:

(18) $$X_{V_{\mathrm{dsDNA}}}=\;X.$$

In the genes of double-stranded RNA viruses $V_{\mathrm{dsRNA}}$, single-stranded RNA viruses $V_{\mathrm{ssRNA}}$, and retro-transcribing viruses $V_{\mathrm{rt}}$, respectively, the nine CP trinucleotide sets $T_{1},\ldots ,T_{4},T_{6},\ldots ,T_{10}\in X$ (except $T_{5}$) have occurrence probabilities $Pb\left(T,V_{\mathrm{dsRNA}}\right)$, $Pb\left(T,V_{\mathrm{ssRNA}}\right)$, and $Pb\left(T,V_{\mathrm{rt}}\right)$, respectively, with the nine highest ranks $Rk\left(T,V_{\mathrm{dsRNA}}\right)$, $Rk\left(T,V_{\mathrm{ssRNA}}\right)$, and $Rk\left(T,V_{\mathrm{rt}}\right)$, respectively, among 30 (Table 2). Note that the ranks $Rk\left(T,V_{\mathrm{dsRNA}}\right)$, $Rk\left(T,V_{\mathrm{ssRNA}}\right)$, and $Rk\left(T,V_{\mathrm{rt}}\right)$ for a given CP trinucleotide set are not identical (Table 2). Thus, by using the reasoning mentioned previously ($T_{15}\notin X$ with $Rk\left(T_{15},V\right)>Rk\left(T_{5},V\right)$ for V in $V_{\mathrm{dsRNA}}$, $V_{\mathrm{ssRNA}}$, and $V_{\mathrm{rt}}$), a subset of X of 18 trinucleotides is observed in $V_{\mathrm{dsRNA}}$, $V_{\mathrm{ssRNA}}$, and $V_{\mathrm{rt}}$:

(19) $$X_{V_{\mathrm{dsRNA}}}=\;X\backslash Y_{V_{\mathrm{dsRNA}}}\;\mathrm{with}\;Y_{V_{\mathrm{dsRNA}}}=\left\{\mathrm{CAG},\mathrm{CTG}\right\},$$

(20) $$X_{V_{\mathrm{ssRNA}}}=X\backslash Y_{V_{\mathrm{ssRNA}}}\;\mathrm{with}\;Y_{V_{\mathrm{ssRNA}}}=\left\{\mathrm{CAG},\mathrm{CTG}\right\},$$

(21) $$X_{V_{\mathrm{rt}}}=X\backslash Y_{V_{\mathrm{rt}}}\;\mathrm{with}\;Y_{V_{\mathrm{rt}}}=\left\{\mathrm{CAG},\mathrm{CTG}\right\}.$$

In the genes of single-stranded DNA viruses $V_{\mathrm{ssDNA}}$, the eight CP trinucleotide sets $T_{1},\ldots ,T_{4},T_{6},\ldots ,T_{9}\in X$ (except $T_{5}$ and $T_{10}$) have occurrence probabilities $Pb\left(T,V_{\mathrm{ssDNA}}\right)$ with the eight highest ranks $Rk\left(T,V_{\mathrm{ssDNA}}\right)$ among 30 (Table 2). Thus, by using the reasoning as previously mentioned ($T_{15}\notin X$ with $Rk\left(T_{15},V_{\mathrm{ssDNA}}\right)>Rk\left(T_{5},V_{\mathrm{ssDNA}}\right)$ and $T_{14}\notin X$ with $Rk\left(T_{14},V_{\mathrm{ssDNA}}\right)>Rk\left(T_{10},V_{\mathrm{ssDNA}}\right)$), a subset of X of 16 trinucleotides is observed in $V_{\mathrm{ssDNA}}$:

(22) $$X_{V_{\mathrm{ssDNA}}}=X\backslash Y_{V_{\mathrm{ssDNA}}}\;\mathrm{with}\;Y_{V_{\mathrm{ssDNA}}}=\left\{\mathrm{CAG},\mathrm{CTG},\mathrm{GTA},\mathrm{TAC}\right\}.$$

All these results show that the circular code X is found almost perfectly in DNA genomes, RNA genomes, double-stranded genomes, and single-stranded genomes. The very few exceptions, either the two trinucleotides {CAG,CTG} or the four trinucleotides {CAG,CTG,GTA,TAC} for one case, are related to the CP set or the two CP sets having the lowest occurrence among the 10 CP sets $T_{1},\ldots ,T_{10}\in X$.

## 4. Conclusions

The “universal” occurrence in genes of a same set X of 20 trinucleotides, which has in addition the mathematical property to be a circular code, must be confirmed by several statistical approaches and various gene data analyses at different levels: kingdom, taxonomic group, genome, and gene. All the previous approaches have studied and identified the circular code X at the gene population level (kingdom, taxonomic group, and genome) [1,2,15,16,17,21]. The statistical approach at the gene level developed here, for the first time since 1996, analyses the preferential occurrence of trinucleotides among the three frames of each gene. This new methodology allows all genes, i.e., of large and small lengths, to be considered with the same weight. As a consequence, the concept of circular code, in particular the reading frame retrieval, is directly associated to each gene. Thus, X motifs from the circular code X at different locations in a gene may assist the ribosome to maintain and synchronize the reading frame. The number, the cardinality, and the length of X motifs in genes may be associated to the length, the function, and the ancestry of genes. This research work is currently under investigation.

At the gene level, the circular code X is strengthened in the genes of bacteria, eukaryotes, plasmids, and viruses, and is now also identified in the genes of archaea. In addition to eukaryotic genomes, it is also found in the genes of eukaryotic chromosomes. The genes of mitochondria and chloroplasts contain a subset of the circular code X. It should be stressed that some mitochondrial and chloroplast genes lack the stop codon and are excluded from this data acquisition. Such a statistical bias may prevent a proper detection of preferential frames for some trinucleotides in the genes of eukaryotic organelles. The circular code X is searched in the large class of $\left(\begin{array}{c} 30\\ 10 \end{array}\right)=30,045,015$ $C^{3}$ self-complementary trinucleotide codes which contains in particular the 216 maximal $C^{3}$ self-complementary circular codes. Thus, for a basic order of magnitude, the probability to retrieve the same circular code X in four independent gene kingdoms (bacteria B, plasmids ℙ, eukaryotes E, double-stranded DNA viruses $V_{\mathrm{dsDNA}}$) is equal to $1/{\left(\begin{array}{c} 30\\ 10 \end{array}\right)}^{4}\approx 10^{-30}$.

In the genes of the bacterial, eukaryotic, and plasmid kingdoms, 14 among the 47 studied gene taxonomic groups (about 30%) have variant X codes [1], i.e., trinucleotide codes which differ from X. Seven variant X codes are identified. However, all have at least 16 trinucleotides of X. Two variant X codes $X_{A}$ (according to the notation in [1]) in cyanobacteria and plasmids of cyanobacteria, and $X_{D}$ in birds, are self-complementary, without permuted trinucleotides, but are non-circular. Five variant X codes $X_{B}$ in *Deinococcus*, plasmids of chloroflexi and *Deinococcus*, mammals, and kinetoplasts, $X_{C}$ in elusimicrobia and apicomplexans, $X_{E}$ in fishes, $X_{F}$ in insects, and $X_{G}$ in basidiomycetes and plasmids of spirochaetes, are $C^{3}$ self-complementary circular. Thus, two variant X codes $X_{A}$ and $X_{D}$ are not circular and do not belong to the set of the 216 maximal $C^{3}$ self-complementary circular codes [2] having the strong mathematical structure of the dihedral group [20]. The reason could be related to the gene data or to a biological property which remains to be identified. All these variant X codes in the genes are identified at the taxonomic group level. However, as the circular code X is now also identified at the gene level, variant X codes may also be associated with genes belonging to the same genome but with different protein coding functions. This interesting and open problem should be investigated in the future.

A probability measure of the reading frame retrieval (RFR) of each trinucleotide of X has been introduced in [22] and [23] (Section 2.2 and 1st row of Table 1). The RFR probability PrRFR of the circular code X, i.e., the average RFR probability of the 20 trinucleotides of X, is equal to PrRFR(X)=82.5% (Result 5 in [22]; 1st row of Table 1 in [23]). This RFR measure can be applied to the non-maximal $C^{3}$ self-complementary circular codes, precisely to the excluded trinucleotides $Y_{A}=\left\{\mathrm{ACC},\mathrm{GGT}\right\}$ of archaea (Equation (11)), $Y_{M}=\left\{\mathrm{AAT},\mathrm{ATC},\mathrm{ATT},\mathrm{CAG},\mathrm{CTC},\mathrm{CTG},\mathrm{GAG},\mathrm{GAT}\right\}$ of mitochondria (Equation (15)), $Y_{\mathbb{C}}=\left\{\mathrm{ATC},\mathrm{CAG},\mathrm{CTC},\mathrm{CTG},\mathrm{GAC},\mathrm{GAG},\mathrm{GAT},\mathrm{GTA},\mathrm{GTC},\mathrm{TAC}\right\}$ of chloroplasts (Equation (16)), $Y_{V}=\left\{\mathrm{CAG},\mathrm{CTG}\right\}$ of viruses (Equation (17)), and $Y_{V_{\mathrm{ssDNA}}}=\left\{\mathrm{CAG},\mathrm{CTG},\mathrm{GTA},\mathrm{TAC}\right\}$ of single-stranded DNA viruses (Equation (22)). The computation leads to $PrRFR\left(Y_{A}\right)=69.0\%$, $PrRFR\left(Y_{M}\right)=88.5\%$, $PrRFR\left(Y_{\mathbb{C}}\right)=87.1\%$, $PrRFR\left(Y_{V}\right)=100.0\%$, and $PrRFR\left(Y_{V_{\mathrm{ssDNA}}}\right)=85.7\%$. Archaeal genes miss two trinucleotides of X which have the lowest RFR values. In contrast, mitochondrial, chloroplast, and viral genes miss trinucleotides of X with high RFR values. Thus, the genes in reduced genomes are more flexible in translation, allowing overlap coding by frameshifting in agreement with [24] (and the cited references). However, it should be stressed that this result may vary with the increase of gene data of eukaryotic organelles in the future. The circular code X (20 trinucleotides) with the functions of reading frame retrieval and maintenance in regular RNA transcription, may also have, through its bijective transformation codes, the same functions in nucleotide exchanging RNA transcription in mitochondrial genes [23]. Indeed, as the mitochondrial gamma polymerase has bacterial origins (e.g., [25]), mitochondrial polymerization and its associated bijective transformations might use the circular code X. However at the translational level, the ribosome might follow the non-maximal $C^{3}$ self-complementary circular code $X_{M}$ observed in mitochondrial genes (Equation (15)). A similar explanation could be applied to the chloroplast genes which have also bacterial origins (cyanobacteria).

By a study of viral genes, the circular code X is found in DNA genomes, RNA genomes, double-stranded genomes, and single-stranded genomes. Thus, the reading frame retrieval property of X could operate for translating DNA and RNA genes, in particular for the “primitive” RNA genes. The $C^{3}$ property of X could be involved for translating the two shifted frames in DNA and RNA genes, in particular for optimizing the genomes of small sizes. The complementarity property of X is naturally associated to the double-stranded DNA and RNA genomes. It could also be used to pair single-stranded DNA genomes between them and single-stranded RNA genomes between them. Thus, the $C^{3}$ and complementary properties of X could be involved for translating the three frames (reading frame and its two shifted frames) in one strand and the three frames in the complementary strand of DNA and RNA genes.

In summary, this new statistical approach at the gene level which is applied to massive gene data identifies the maximal $C^{3}$ self-complementary trinucleotide circular code X in the genes of bacteria, archaea, eukaryotes, plasmids, and viruses, which may be involved in translation coding [3].

## Figures and Tables

**Table 1:** Kingdoms K of genes extracted from the GenBank database (http:// www.ncbi.nlm.nih.gov/genome/browse/, May 2016) with their symbol and their numbers of genomes, genes, and trinucleotides.

Kingdom	K (Symbol)	Nb of Genomes	Nb of Genes	Nb of Trinucleotides
Bacteria	B	7039	15,735,053	5,222,267,667
Archaea	A	182	282,802	81,460,549
Plasmids	ℙ	2319	575,760	159,169,387
Eukaryotes	E	190	4,356,391	2,406,844,838
Chromosomes of eukaryotes	$E_{\mathrm{chr}}$	2979	4,356,391	2,406,844,838
Mitochondria	M	228	3347	862,327
Chloroplasts	ℂ	39	3192	925,303
Viruses	V	5217	299,401	66,677,580
Double-stranded DNA viruses	$V_{\mathrm{dsDNA}}\subset V$	2480	259,696	59,239,700
Double-stranded RNA viruses	$V_{\mathrm{dsRNA}}\subset V$	211	1061	783,020
Single-stranded DNA viruses	$V_{\mathrm{ssDNA}}\subset V$	715	3291	802,405
Single-stranded RNA viruses	$V_{\mathrm{ssRNA}}\subset V$	1257	5093	4,406,365
Retro-transcribing viruses	$V_{\mathrm{rt}}\subset V$	137	560	289,447

**Table 2:** Identification of the maximal $C^{3}$ self-complementary trinucleotide circular code X in gene kingdoms K of bacteria B, archaea A, plasmids ℙ, eukaryotes E, chromosomes of eukaryotes $E_{\mathrm{chr}}$, mitochondria M, chloroplasts ℂ, viruses V, and its five taxonomic groups: double-stranded DNA viruses $V_{\mathrm{dsDNA}}$, double-stranded RNA viruses $V_{\mathrm{dsRNA}}$, single-stranded DNA viruses $V_{\mathrm{ssDNA}}$, single-stranded RNA viruses $V_{\mathrm{ssRNA}}$, and retro-transcribing viruses $V_{\mathrm{rt}}$ (Table 1). Occurrence probability Pb(T,K) (%) of the 30 complementary and permutation (CP) trinucleotide sets $T=\left\{T^{0},T^{1},T^{2}\right\}$ with $T^{0}=\left\{t,C\left(t\right)\right\}$ in frame 0, $T^{1}=P(T^{0})=\left\{P\left(t\right),P(C\left(t\right))\right\}$ in frame 1, $T^{2}=P^{2}(T^{0})=\left\{P^{2}\left(t\right),P^{2}(C\left(t\right))\right\}$ in frame 2, in a gene kingdom K computed according to Equation (9) and its rank Rk(T,K), the 1st rank being associated to the highest value of Pb(T,K) and the 30th rank, to the lowest value of Pb(T,K). The 20 trinucleotides of the $C^{3}$ self-complementary circular code X are in bold, the 20 trinucleotides of the circular code $X_{1}=P\left(X\right)$ are in italics, and the 20 trinucleotides of the circular code $X_{2}=P^{2}\left(X\right)$ are both in bold and italics. The first 10 CP sets $T_{1},\ldots ,T_{10}$ belong to the circular code X ($T^{0}=\left\{t,C\left(t\right)\right\}\in X$ with $T^{1}=P(T^{0})\in P\left(X\right)=X_{1}$ and $T^{2}=P^{2}(T^{0})\in P^{2}\left(X\right)=X_{2}$) and are in lexicographical order with respect to the trinucleotide t∈X in bold, and the 20 remaining CP sets $T_{11},\ldots ,T_{30}$ are in lexicographical order with respect to the trinucleotide $t\in X_{1}$ in italics. The numbers in italics occurring with the CP sets $T_{1},\ldots ,T_{10}$ are associated with the two trinucleotides $T^{0}=\left\{t,C\left(t\right)\right\}$ of X which do not occur preferentially in the gene kingdom.

			B		A		ℙ		E		$E_{chr}$		M		ℂ		V		$V_{\mathrm{dsDNA}}$		$V_{\mathrm{dsRNA}}$		$V_{\mathrm{ssDNA}}$		$V_{\mathrm{ssRNA}}$		$V_{\mathrm{rt}}$	
T	t	C(t)	Pb	Rk	Pb	Rk	Pb	Rk	Pb	Rk	Pb	Rk	Pb	Rk	Pb	Rk	Pb	Rk	Pb	Rk	Pb	Rk	Pb	Rk	Pb	Rk	Pb	Rk
$T_{1}$	**AAC**	**GTT**	6.1	6	7.4	4	6.0	4	8.5	3	8.4	3	4.4	9	4.9	9	6.6	3	6.8	4	6.4	3	5.9	1	7.0	3	5.0	5
$T_{2}$	**AAT**	**ATT**	7.4	2	5.3	8	7.3	2	8.7	2	8.7	2	*3.4*	*14*	5.8	1	6.6	2	7.5	2	5.9	4	5.6	2	6.3	4	5.3	4
$T_{3}$	**ACC**	**GGT**	5.1	9	*3.9*	*12*	5.1	8	5.1	8	4.7	10	5.7	4	5.2	3	4.9	8	4.9	9	4.6	8	4.6	5	5.3	7	3.7	9
$T_{4}$	**ATC**	**GAT**	6.5	4	6.7	5	6.2	3	8.1	4	8.2	4	*3.6*	*13*	*4.6*	*14*	6.5	4	6.7	5	6.4	2	5.4	4	7.1	2	6.0	2
$T_{5}$	**CAG**	**CTG**	4.6	10	3.7	13	3.9	10	4.2	10	4.9	9	*0.7*	*30*	*0.0*	*30*	*2.9*	*15*	3.8	10	*2.4*	*18*	*2.8*	*19*	*1.5*	*24*	*2.9*	*18*
$T_{6}$	**CTC**	**GAG**	6.2	5	7.5	3	5.9	6	7.0	5	7.5	5	*2.6*	*18*	*0.4*	*27*	5.6	6	6.3	6	5.3	7	4.1	10	5.4	6	4.9	6
$T_{7}$	**GAA**	**TTC**	5.8	7	5.3	7	5.5	7	5.0	9	5.2	7	6.3	2	4.9	7	5.2	7	5.6	7	5.3	6	4.6	6	4.9	8	5.4	3
$T_{8}$	**GAC**	**GTC**	8.2	1	9.7	1	7.8	1	9.0	1	9.1	1	5.8	3	*0.6*	*26*	7.2	1	8.0	1	7.3	1	5.4	3	7.3	1	6.4	1
$T_{9}$	**GCC**	**GGC**	6.7	3	8.2	2	6.0	5	5.7	6	5.2	8	7.1	1	5.3	2	5.9	5	7.0	3	5.5	5	4.3	8	5.7	5	4.7	7
$T_{10}$	**GTA**	**TAC**	5.4	8	6.6	6	5.0	9	5.4	7	5.7	6	4.6	8	*4.8*	*11*	4.7	9	5.3	8	4.6	9	*4.0*	*11*	4.5	10	4.3	8
$T_{11}$	*AAG*	***CTT***	3.0	13	4.1	11	2.9	16	3.4	14	3.5	13	1.4	27	3.2	18	3.3	12	3.0	14	3.2	13	3.6	12	3.6	13	2.9	16
$T_{12}$	*ACA*	***TGT***	1.1	26	1.4	20	1.1	26	0.3	30	0.2	30	4.3	10	2.9	19	1.2	27	0.9	26	1.3	28	1.4	28	1.5	26	1.5	29
$T_{13}$	*ACG*	***CGT***	1.6	22	0.3	28	1.8	21	0.6	26	0.6	25	1.9	24	5.0	4	1.8	22	1.4	23	1.6	25	3.2	16	1.6	22	2.1	24
$T_{14}$	*ACT*	***AGT***	2.9	15	1.3	22	3.3	13	3.0	15	2.5	15	2.1	22	4.9	8	3.6	11	3.1	13	3.7	11	4.3	7	4.1	11	3.4	14
$T_{15}$	*AGC*	***GCT***	2.9	14	3.4	14	3.1	15	3.4	13	3.1	14	3.9	12	4.9	6	4.0	10	3.6	11	4.2	10	4.3	9	4.6	9	3.5	13
$T_{16}$	*AGG*	***CCT***	2.3	19	1.5	19	2.5	19	2.4	16	2.0	17	2.2	21	4.8	13	2.7	17	2.5	17	2.6	17	3.5	13	2.7	17	2.4	22
$T_{17}$	*ATA*	***TAT***	1.6	21	4.1	10	1.6	24	0.8	23	0.9	23	4.2	11	2.7	20	2.3	19	1.7	20	2.9	16	2.8	21	2.7	16	2.9	17
$T_{18}$	*ATG*	***CAT***	3.1	12	2.5	16	3.2	14	1.3	20	1.4	19	1.8	26	4.8	10	2.5	18	2.6	15	2.3	19	3.1	17	1.8	20	2.7	20
$T_{19}$	*CCA*	***TGG***	1.6	23	1.3	21	1.8	20	1.1	22	0.8	24	2.8	16	4.5	15	2.0	21	1.6	22	2.3	20	2.6	22	1.8	19	2.8	19
$T_{20}$	*CCG*	***CGG***	0.6	28	0.1	29	0.7	28	0.8	24	1.0	22	0.8	29	0.6	25	1.5	26	0.8	28	1.4	27	2.8	20	1.5	25	2.3	23
$T_{21}$	*GCG*	***CGC***	2.7	17	1.7	18	3.4	11	3.5	12	3.9	12	2.0	23	4.2	17	2.9	16	2.4	18	3.2	15	3.4	14	3.0	14	3.6	12
$T_{22}$	*GTG*	***CAC***	3.3	11	4.7	9	3.4	12	3.8	11	4.5	11	1.8	25	0.3	28	3.3	13	3.5	12	3.2	14	3.2	15	2.9	15	3.7	10
$T_{23}$	*TAG*	***CTA***	1.7	20	2.1	17	1.6	22	1.6	19	1.8	18	3.0	15	0.3	29	1.7	24	1.6	21	2.1	22	1.8	26	1.6	23	2.0	25
$T_{24}$	*TCA*	***TGA***	0.4	29	0.8	25	0.6	29	0.6	25	0.4	28	4.8	7	0.7	24	0.7	30	0.6	30	1.0	29	0.9	30	0.8	30	0.9	30
$T_{25}$	*TCC*	***GGA***	1.5	24	1.0	24	1.6	23	0.6	27	0.6	26	5.0	6	4.8	12	1.7	23	1.1	25	1.9	23	2.4	23	2.0	18	2.7	21
$T_{26}$	*TCG*	***CGA***	0.2	30	0.1	30	0.4	30	0.4	29	0.3	29	2.6	17	4.3	16	1.0	29	0.7	29	0.8	30	1.7	27	1.0	28	1.7	28
$T_{27}$	*TCT*	***AGA***	1.2	25	0.6	27	1.5	25	1.6	18	1.3	21	2.4	19	2.1	22	1.6	25	1.4	24	1.8	24	2.0	25	1.7	21	1.7	27
$T_{28}$	*TGC*	***GCA***	2.5	18	3.0	15	2.8	18	2.4	17	2.0	16	5.2	5	4.9	5	3.0	14	2.6	16	3.4	12	2.9	18	3.7	12	3.2	15
$T_{29}$	*TTA*	***TAA***	0.9	27	0.6	26	1.0	27	0.5	28	0.4	27	2.3	20	1.6	23	1.1	28	0.9	27	1.5	26	1.4	29	1.0	29	1.9	26
$T_{30}$	*TTG*	***CAA***	2.8	16	1.2	23	2.9	17	1.2	21	1.4	20	1.2	28	2.3	21	2.1	20	2.2	19	2.2	21	2.3	24	1.4	27	3.6	11
